# C-type natriuretic peptide prevents activation of perivascular mast cells and inflammation in the postischemic microvasculature

**DOI:** 10.1186/2050-6511-16-S1-A41

**Published:** 2015-09-02

**Authors:** Wen Chen, Katharina Völker, Birgit Gaßner, Franziska Werner, Anja Rabenhorst, Karin Hartmann, Michaela Kuhn

**Affiliations:** 1 Institute of Physiology, University of Würzburg, Würzburg, Germany; 2 Department of Dermatology, University of Cologne, Cologne, Germany

## Background

Atrial- and C-type- natriuretic peptides (ANP, CNP) are both vasodilatatory hormones which are mainly secreted from cardiac atria and vascular endothelium, respectively. Both peptides activate cyclic GMP (cGMP) formation by binding to their specific receptors: the particulate guanylyl cyclases A (GC-A) and B (GC-B). Cyclic GMP via cGMP-dependent protein kinase I (cGKI) was shown to inhibit mast cell (MC) degranulation [1]. During postischemic reperfusion, the activation of MC is detrimental [2]. Due to pleiotropic effects, cytokines released from MC cause tissue damage and vascular leakage. It is unclear whether and how ANP and/or CNP regulate the activity of MC and, thereby, influence ischemia-reperfusion-induced disruption of the microvascular barrier.

## Methods and results

Intravital microscopy was used to visualize the microcirculation within the cremaster muscle of anesthetized mice, and Fluorescein isothiocyanate (FITC)-Dextran was injected intravenously to quantify the microvascular leakage [3,4]. Cremaster blood flow was temporally blocked with an arterial clamp to provoke 30 min of tissue ischemia. During the following 30 min of postischemic reperfusion, the vascular leakage was measured by the ratio of the fluorescent (FITC-Dextran) intensity (RFI) between the adjacent interstitial space and the intravascular lumen. Subsequently resident perivascular degranulated MCs were stained by superfusing Ruthenium Red solution and thereafter counted (number per field).

In wildtype mice, ischemia-reperfusion (compared to sham operated cremaster muscles) provoked marked MC degranulation and robust vascular leakage of FITC-Dextran (as indicated by higher RFI). Local CNP superfusion during reperfusion significantly reduced the amount of degranulated MCs and concomitantly vascular leakage. These effects were mimicked by the membrane-permeable cGKI activator 8-Br-cGMP. The protective effects of 8-Br-cGMP were endothelium-independent, since they were preserved in mice with conditional, endothelial deletion of cGKI. Quantitative real-time RT-PCR revealed local CNP-mRNA expression in the m. cremaster, which was not altered by acute ischemia. In contrast to CNP, ANP superfusion did not significantly alter the amount of degranulated MCs, but still effectively reduced FITC-Dextran leakage resulted from ischemia-reperfusion. Accordingly, in vitro, in cultured human MCs (HMC-1.1, originally developed by Dr. Butterfield and a gift from Dr. Drube) and primary cultured murine bone marrow MC, CNP, but not ANP, induced a marked increase in intracellular cGMP levels and enhanced the phosphorylation of the cytoskeleton-associated vasoactive stimulated phosphoprotein (VASP) at Ser237.

## Conclusions

Our study shows divergent effects of NPs in the postischemic microcirculation. CNP inhibited the ischemic activation of resident, perivascular MCs and consequently the associated microvascular leakage, possibly via MC specific cGMP/cGKI signaling. At difference, ANP had no effect on MCs but still attenuated the postischemic vascular barrier disruption via direct effects on endothelial cells. Our future studies aim to dissect the vascular cell-specific effects and signaling pathways of both NPs and possible clinical implications.
